# Assessment of the Anti-*Listeria* Effect of *Citrus limon* Peel Extract In Silico, In Vitro, and in Fermented Cow Milk During Cold Storage

**DOI:** 10.3390/foods14040661

**Published:** 2025-02-15

**Authors:** Wafa Mkadem, Khaoula Belguith, Valentina Indio, Olfa Oussaief, Gulnara Guluzade, Halima ElHatmi, Andrea Serraino, Alessandra De Cesare, Nourhene Boudhrioua

**Affiliations:** 1 Laboratory of Physiopathology, Alimentation and Biomolecules (LR17ES03), Higher Institute of Biotechnology Sidi Thabet, University of Manouba, Ariana BP-66, Manouba 2020, Tunisia; 2 Department of Veterinary Medical Sciences, Alma Mater Studiorum-University of Bologna, Via Tolara di Sopra 50, 40064 Ozzano dell’Emilia, BO, Italy; 3 Livestock and Wildlife Laboratory, Arid Lands Institute of Medenine, University of Gabes, Medenine 4119, Tunisia; 4 Food Department, Higher Institute of Applied Biology of Medenine, University of Gabes, Medenine 4119, Tunisia

**Keywords:** *Citrus limon* peel extract, *Listeria monocytogenes*, docking visualization, fermented milk

## Abstract

In this study, the antimicrobial effect of *Citrus limon* peel extract against *Listeria monocytogenes* was analyzed in silico, tested in vitro, and validated in fermented cow milk during cold storage. The in silico analysis revealed that 4,5-di-O-caffeoyquinic acid interacts with *L. monocytogenes* proteins involved in colonization and intracellular survival. The in vitro experiments demonstrated that the anti-*Listeria* activity of *Citrus limon* peel extract is primarily attributed to limonene and phenolic compounds. In fermented milk stored at 4 °C for 7 days, the addition of *Citrus limon* peel extract resulted in a 2 Log reduction of *L. monocytogenes* compared to the control. Using the Baranyi and Roberts model, a significant decrease in the maximum growth rate (−0.021 h^−1^) and the concentration of *L. monocytogenes* from 5.95 to 3.67 log CFU/mL was observed in fermented milk supplemented with a 2×MIC level of *Citrus limon* peel extract during storage at 4 °C. The findings from all three approaches highlighted that the inhibitory effect of *Citrus limon* peel extract against *L. monocytogenes* is primarily due to chlorogenic acid derivatives, especially 4,5-di-O-caffeoyquinic acid, and limonene. Beyond its antimicrobial properties, the supplementation of fermented milk with *Citrus limon* peel extract also enhances the milk antioxidant capacity and total organic acids content.

## 1. Introduction

*Listeria monocytogenes* is one of the most severe zoonotic diseases, characterized by the highest hospitalization rates among cases and the highest case fatality rates [1]. It is monitored throughout the food chain, including primary production, manufacturing, and distribution stages. In 2023, 27 European Member States reported 2952 confirmed cases of invasive *L. monocytogenes* infections in humans, resulting in a European Union notification rate of 0.66 cases per 100,000 population [1]. This represents a 5.8% increase compared to the 2022 rate of 0.63 cases per 100,000 population and marks the highest rate and number of cases reported since 2007 [1].

A wide range of substances are effective against *L. monocytogenes*, including essential oils [2], bacteriocins such as nisin [3], and bacteriophages [4]. The choice of anti-*Listeria* strategy depends on the food product, processing environment, and regulatory guidelines. In this study we investigated the anti-*Listeria monocytogenes* effect of *Citrus limon* peel extract (LPE), assessing its biological effect in silico, testing its inhibitory efficacy at different concentrations in vitro, and validating its effect in vivo in fermented cow milk.

*Citrus limon* peel is the rind of lemons and represents a co-product of lemon processing. *Citrus* peel is mainly composed of vitamins (65 mg/g for vitamin C), fiber (86 mg/g) (613.11 ± 1.19 mg/100 g for potassium), and particularly, polyphenolic compounds (179 mg/g). Beyond its refreshing flavor and unique sensory properties, *Citrus limon* peel has been studied for its bioactivity potential, largely due to its abundance of antioxidant compounds [5,6]. Citrus phenolic acids, flavonoids, and limonene are the primary compounds of *Citrus limon* peel, demonstrating significant potential in inhibiting spoilage and pathogenic microorganisms, as well as preventing food oxidation [5].

The antimicrobial effects of phenolic compounds are primarily attributed to the damage of cell membranes and inhibition of the synthesis of nucleic acids and proteins [7]. Extensive research has been focused on investigating the interaction between bioactive compounds and target protein receptors in the membranes of foodborne pathogens. In this context, molecular docking has emerged as an efficient and cost-effective computational approach to design and elucidate interactions between structures involved in anti-microbial processes [8]. Numerous studies have evaluated the functional properties of *Citrus limon* peel derivatives in products such as yogurt [9], ice cream [10], kefir [11], and cheese [12]. However, these studies mainly focused on sensory and physicochemical effects. To the best of our knowledge, the anti-*Listeria* effect of the phenolic extracts from *Citrus limon* peel have not been studied in dairy products, particularly in fermented cow milk.

The risk of contamination of dairy products with *L. monocytogenes* primarily arises from environmental sources, where the pathogen persists due to inadequate zoning and hygiene barriers, poor hygienic design of equipment and machinery, and insufficient cleaning and disinfection practices [13]. Despite the low prevalence in ready-to-eat (RTE) dairy products, *L. monocytogenes* can grow during cold storage, potentially reaching levels significantly higher than 100 CFU/g during the consumer phase due to inappropriate storage and handling [14,15].

The use of plant-based bio-preservatives offers a sustainable alternative to thermal processing to reduce *L. monocytogenes* in dairy products while also enhancing the dairy product’s antioxidant potential and total organic acids content [16]. Therefore, in this study, the antimicrobial effect of *Citrus limon* peel extract against *Listeria monocytogenes* was analyzed in silico, tested in vitro, and validated in fermented cow milk during cold storage.

## 2. Materials and Methods

### 2.1. Preparation of Citrus limon Peel Extract (LPE)

A total of 7 kg of lemons were sourced from a local market in Northern Tunisia. The fruits were washed thoroughly, and the peels (approximately 2 kg) were manually removed, with any blemished or damaged parts discarded. The peels were dried at 60 °C in a laboratory electric thermostatic oven (Memmert GmbH + Co. KG Universal Oven UN30; Schwabach, Germany) until a constant weight was achieved. The dried peels were then ground using a blender (Moulinex SA, Bagnolet, France) and sieved with a 500 μm mesh sieve. Solvent extraction was carried out using an 80 % ethanol (Sigma Aldrich)/water ratio (*v*/*v*) and a powder/solvent volume ratio of 1:10 (*w*/*v*) at 40 °C for 30 min at a rotational speed of 200 rpm using an orbital shaker (Heavy Duty Shaker, OHAUS™, Parsippany, NJ, USA) [17]. The supernatants from three extractions were pooled and filtered through Whatman filter paper no. 1 (pore size: 11 μm). Ethanol was evaporated using a rotary evaporator (Stuart, RE300, Germany), and the resulting aqueous extract was freeze-dried using a freeze dryer (Biobase, BK-FD12P, Jinan, China). The freeze-dried extract was stored at 5 °C until further analysis.

### 2.2. Phytochemical Characterization of Citrus Limon Peel Extract and Antioxidant Activity

#### 2.2.1. Determination of Total Phenol and Flavonoid Contents and Antioxidant Activity

Total phenol and flavonoid contents and free radical scavenging activity were evaluated for lemon peel extract as described by Mhiri et al. [6] and Ben Abdallah et al. [18].

The total phenol content (TPC) of LPE was evaluated using the Folin–Ciocalteu assay. Specifically, 40 µL of citrus extract was mixed with 200 µL of Folin–Ciocalteu reagent and 3.160 mL of distilled water. The mixture was then stirred for 3 min at room temperature, followed by the addition of 600 µL of a 20% Na_2_CO_3_ solution. The resulting solution was incubated at 40 °C for 30 min, after which the absorbance was measured at 765 nm using a UV spectrophotometer (UV-6300PC, VWR International, LLC, Radnor PA, USA). The results were expressed as g gallic acid equivalent (GAE) per 100 g of the freeze-dried extract. The aluminum chloride colorimetric method was adopted for the total flavonoid content (TFC) measurements. Citrus extract (500 µL) was mixed with 2.5 mL distilled water and 150 µL NaNO_2_ (5%). After 5 min, 150 µL of AlCl_3_ was added and the mixture was allowed to stand for 5 min. Then, 1 mL of NaOH (1M) was added and the final volume was adjusted to 5 mL with distilled water. The total flavonoid content (TFC) results were given according to the quercetin (QE) concentration range (0–500 mg/mL) and expressed as g QE/100 g of extract. The radical scavenging capacity was determined by 1.1-diphenyl-2-picrylhydrazyl (DPPH) radical scavenging and 2,2’-azino-bis (3-ethylbenzothiazoline-6-sulphonic acid) (ABTS) assays. For the DPPH test, 400 µL of extract was mixed with 2.4 mL of DPPH solution (63.4 µM) and incubated in the dark for 30 min. The scavenging capacity was determined at 515 nm, and the obtained results were expressed as mg Trolox Equivalent (0–125 mg/mL) per gram. For the ABTS assay, lemon peel extract (50 µL) was mixed with ABTS solution prepared by dissolving 7 mM of ABTS stock solution in 3 mM potassium persulfate (K_2_S_2_O_3_). ABTS antioxidant activity was measured at 734 nm and expressed as mg Trolox (0–250 µg/mL) equivalent per g.

#### 2.2.2. Determination of Aromatic Compounds

Aromatic compounds of citrus peel powder were detected by gas chromatography QP2010 Shimadzu using an RTX-5MS capillary column (30 m × 0.25 mm i.d. and 0.25 µm film thickness) (Bellefonte, PA, USA). The column oven temperature was maintained at 45 °C. The injection temperature was 250 °C in split mode. The total flow rate was 5 mL/min, and the column flow rate was 1 mL/min as indicated by Jrad et al. [19].

#### 2.2.3. Identification of Individual Phenolic Compounds

Individual phenolic compounds were quantified using a Shimadzu LC–MS− 2020 system (Shimadzu, Milan, Italy). The system was equipped with SPD–M20A photodiode array (PDA) detector, a quadrupolar mass analyzer, and electrospray (ESI) with atmospheric pressure chemical ionization (APCI). Separation was performed on an AQUASIL C18 column (150 mm × 3.0 mm, 3 µm), and conditions were adopted according to El Hatmi et al. [20].

### 2.3. In Silico Simulation and Visualization of Anti-Listeria Effects

A molecular docking study was performed according to the methodology adopted by Noshad et al. [8] using the predominant phyto-compounds, which included two phenolic compounds and two volatile ones of the LPE identified by LC-MS and GC-MS analyses. These compounds were chosen as ligands and tested against six target *L. monocytogenes* proteins. The studied proteins included internalin C (PDB ID: 1XEU), internalin B (InlB) (PDB ID: 2WQV), internalin K (InlK) (PDB ID: 4L3A), internalin-like protein Lmo2027 (PDB ID: 5KZS), Regulator of MurA (ReoM) (PDB ID: 6TIF), and PadR-like protein (PDB ID: 7WJP). These proteins are involved in various mechanisms of *L. monocytogenes* virulence, including invasion, cell wall homeostasis, and survival in extreme environments, such as acidic conditions or in the presence of bile. The structures of the selected ligands and receptors were retrieved from the Public Chemical Database (PubChem) (https://pubchem.ncbi.nlm.nih.gov/) and the RCSB Protein Data Bank (https://www.rcsb.org/), respectively. Molecular docking was conducted using the AutoDock Vina function implemented in PyRx v0.8 virtual screening software, employing a blind docking approach. The docking parameters for each protein are provided in Table S1. Ligand–protein complex interactions were visualized using Discovery Studio (Dassault Systèmes BIOVIA, San Diego, CA, USA) for both 2D and 3D interactions.

### 2.4. In Vitro Anti-Listeria Activity of Citrus limon Peel Extract

The minimum inhibitory concentration (MIC) of LPE was assessed against *L. monocytogenes* (*ATCC 19117*) according to the method described by Ben Hsouna et al. [21]. The broth microdilution method was used with LPE concentration varied from 200 to 6.25 mg/mL. The bacterial suspension (10 µL) was added at 0.5 McFarland concentration in each well. The microtiter plates were incubated at 37 °C for 24 h, and the microbial growth was controlled using the MTT indicator (0.5 mg/mL).

Different concentrations of LPE were prepared corresponding to 1×MIC, 2×MIC, and 4×MIC in brain heart infusion broth in microtiter plates and evaluated during *L. monocytogenes* growth according to Sharma et al. [22] with some modifications. Bacterial suspension of *L. monocytogenes* was added into wells at a 1:1 ratio. The microtiter plates were then incubated at 37 °C for 24 h and the optical density was measured every 2 h at 600 nm using a Multiskan spectrophotometer (Thermo Scientific, Vantaa, Finland).

Each optical density was determined in triplicate and results were subjected to an analysis of variance (ANOVA) using XLSTA software version 2019 (Addinsoft, Paris, France).

### 2.5. Anti-Listeria Potential of Lemon Peel Extract in Fermented Milk During Storage

#### 2.5.1. Fermented Milk Preparation

Raw cow milk was collected from the experimental farm of the Department of Veterinary Medical Sciences of the University of Bologna (Italy). Milk was pasteurized at 80 °C for 5 min and then fermented using *L. paracasei OWS23*. This strain, an autochthonous bacterium isolated from fermented milk, was previously characterized by Mkadem et al. [23]. *L. paracasei* is classified as Generally Recognized as Safe (GRAS) and has the Qualified Presumption of Safety (QPS) status, making it suitable for inclusion in food products [24]. In our previous study [23], genomic analysis revealed critical insights into the strain’s antimicrobial features, particularly the presence of genes responsible for bacteriocins production (e.g., LSEI 2386) and bacteriocin immunity protein.

For the fermentation process, *L. paracasei* from the activated subculture was added to the milk at a concentration of 1% *v*/*v* concentration (10 mL/L), achieving an initial count of approximately 7 log CFU/mL. Fermentation was carried out in different sterilized 1 L bottles at 37 °C for 24 h.

#### 2.5.2. Preparation and Inoculum of *L. monocytogenes*

Prior to inoculation into fermented milk, *L. monocytogenes* was activated twice at 37 °C for 16–18 h. A subculture was performed and the culture was incubated at 4 °C for three days to adapt the bacteria to the storage temperature conditions. The fermented milk samples (Section 2.5.1) were divided into four groups: fermented milk; fermented milk containing LPE at 2×MIC level; fermented milk containing *L. monocytogenes* (LM); fermented milk containing LM and LPE at 2×MIC level.

The LPE was aseptically added to the fermented milk and the samples were homogenized to ensure uniform distribution. For the samples of fermented milk with LM, the bacteria were inoculated directly using a suspension prepared according to EURL Lm Technical Guidance [25] to reach approximately 5 log CFU/mL. All samples were thoroughly mixed to ensure homogeneous microbial distribution and efficient LPE exposure using a vortex (VORTEX 3, IKA, Staufen, Germany). The samples were stored at 4 °C for 7 days before being analyzed as described in Table S2.

#### 2.5.3. Fitting of *L. monocytogenes* Growth Kinetics

Weibull and Baranyi and Roberts models (with no lag phase) were used to fit the growth of *L. monocytogenes* studied in fermented milk during storage. The Weibull model represented in Equation (1) was fitted to experimental data using Matlab@ software (Version 7) to determine kinetic parameters (initial value, time required for first tenfold reduction, and curvature parameter):

(1) $$logN(t)=logN_{0}-({\frac{t}{\delta })}^{\beta }$$

In the equation, *N*(*t*) is the number of surviving cells (CFU/mL); *N*_0_ is the initial number of cells (CFU/mL); δ the time required to have a first tenfold reduction and *β* is the shape parameter (dimensionless).

The Baranyi and Roberts model (Equations (2) and(3)) without lag phase was also used for fitting using the DMFit MS Excel add-in (Food Safety Centre, Hobart, Australia) (https://www.combase.cc) according to Posada-Izquierdo et al. [26].

For this model, four main parameters were determined (initial value, maximum growth rate, final value, and the average of total reduction).

(2) $$y\;\left(t\right)=y_{0}+{µ}_{max}A\left(t\right)-ln(1+(\frac{e^{{µ}_{max}A\left(t\right)}-1}{e^{\left(ymax-y0\right)}})$$

(3) $$A\left(t\right)=t+\left(\frac{1}{{µ}_{max}}\right)ln(e^{-{µ}_{max}t}+e^{-{µ}_{max}\lambda }-e^{-{µ}_{max}(t+\lambda )})$$

where *y*(*t*) represents the bacterial concentration (log CFU/mL) at time t; *y*_0_ is the initial number of cells (log CFU/mL); *y_max_* is the maximum cell concentration (log CFU/mL); μ*_max_* is the maximum growth rate (or maximum death rate in the case of a survival curve) in h^−1^, and λ is the lag phase period (h).

The goodness of fit of both models was evaluated using root mean square error (RMSE) and the coefficient of correlation (R^2^).

#### 2.5.4. Microbiological Analysis

The counts of *L. monocytogenes*, lactic acid bacteria, and total mesophilic bacteria were determined in fermented milk samples in three different batches after sample preparation (time zero) and during 7 days of storage at 4 °C. The enumeration of *L. monocytogenes* was performed according to the ISO 11290-2 [27] using chromogenic Listeria agar Thermo Scientific™ Oxoid™ (Waltham, MA, USA). Plates were incubated at 37 °C for 48 h. MRS agar (Biolife, Italiana S.r.l., Milano, Italy) was used to quantify lactic acid bacteria according to the ISO 15214 [28] method. Total bacteria were counted on Plate Count Agar (PCA, CM0325, Oxoid, Ltd.) according to the ISO 4833-2 [29]. Plates were incubated at 37 °C for 24–48 h.

#### 2.5.5. Measurement of pH and Acidity

The pH and acidity analyses were performed according to Mkadem et al. [30] in fermented milk samples without *L. monocytogenes*. The pH was determined by directly immersing the pH meter electrode (OHAUS starter 2100, Pine, Brook, NJ, USA) and acidity was measured by titration against a 0.1 M sodium hydroxide solution.

#### 2.5.6. Antioxidant Activity and Organic Acids Profile of Fermented Milk Samples

Fermented milk samples were centrifuged at 6000 rpm for 30 min at 4 °C, and the supernatants were filtered using a 0.45 µm syringes filter. Total flavonoid and phenol contents and antioxidant activities were determined for fermented milk (time zero) and during storage, as described for LPE. Organic acids in raw milk and fermented milk samples were quantified using HPLC (Shimadzu UFLC XR), according to Dursun et al. [31]. Briefly, fermented milk samples were mixed with 10 mM sulfuric acid at a ratio of 4:1 (*v*/*v*), and then centrifuged at 6000 rpm for 20 min at 4 °C. The supernatants were filtered through a 0.45 μm syringe filter before HPLC injection. The separation was performed using an Agilent Hi-Plex H (7.7 × 300 mm, 7.8 μm) column. Oxalic, citric, malic, succinic, lactic, formic, acetic, propionic, and butyric acids were quantified in the samples at 210 nm using the linear calibration curves of each organic acid.

### 2.6. Statistical Analysis

Each experiment was performed in triplicate and results are expressed as the mean value. Results were subjected to an analysis of variance (ANOVA) using XLSTAT software version 2019 (Addinsoft, Paris, France).

## 3. Results and Discussion

### 3.1. LPE Bioactivities

#### Total Phenol and Flavonoid Contents, Antioxidant Activity and Phytochemical Composition

The TPC and TFC of LPE were, respectively, 2.32 ± 0.05 g GAE/100 g and 1.71 ± 0.03 g QE/100 g. The extract displayed an interesting radical scavenging capacity (10.18 ± 0.04 mg TE/g by DPPH assay and 122.88 ± 2.09 mg TE/g by ABTS assay). Saleem et al. [32] reported similar phenol and flavonoid contents for a Tunisian ethanolic extract of *Citrus limon* peel. A total of 11 volatile compounds and 15 phenolic compounds were identified in the extract analyzed by GC-MS and LC-MS, respectively. The obtained results showed that limonene and β-pinene were the main volatiles found in LPE dried powder. Lemon, orange, pine, resin, and turpentine-like odors were reported for these compounds (Table 1).

Tekgül and Baysal [33] have reported similar results for the abundance of limonene and β-pinene in *Citrus limon* with a relative abundance of 70.75% for limonene and 13.19% for β-pinene.

Table 2 shows the different phenolic compounds of the *Citrus limon* ethanolic extract. The LPE was dominated by chlorogenic acid derivates, and the most abundant were 4.5-di-O-caffeoylquinic acid (10.9 ± 0.3 mg/g) and quinic acid (3.3 ± 0.1 mg/g). Flavonoid compounds, mainly rutin, hyperoside, and naringin, were also identified in the ethanolic extract. Other phenolic compound derivatives were also identified at low concentrations, primarily hydroxycinnamic acids, such as p-coumaric, o-coumaric, and trans-ferulic acids (0.0133- 0.129- 0.0028 mg/g, respectively), and flavone derivatives, mainly naringenin, cirsiliol, cirsilineol, and epicatechin. The identified phenolic compounds in LPE and the richness of extract in quinic acid with hydroxyl groups (-OH) characterized LPE as a functional ingredient with antioxidant, antibacterial, and antifungal activities [34].

### 3.2. Binding Affinity of Selected Phenolic and Volatiles Compounds Against L. monocytogenes Proteins as Determined in Silico

The major phytochemical compounds in the LPE were selected for molecular docking analysis, displaying variable interactions with the target proteins. The binding energy results are summarized in Table 3, ranging from −7.3 to −4.2 kcal/mol. Among all of the compounds, 4,5-di-O-caffeoyquinic acid emerged as the most potent compound, demonstrating strong interactions with the selected target proteins. The highest binding energy was observed with *L. monocytogenes* internalin-like protein lmo2027 (5KZS), a key protein involved in bacterial colonization and intracellular survival [35]. In contrast, the volatile compound β-Pinene exhibited the lowest binding energy against the same protein (5KZS). Understanding the interactions between ligands and the receptor’s active site is crucial for investigation binding mechanisms and assessing the functional properties of ligands [36].

Moderate binding scores were observed for quinic acid and limonene. The best affinity of quinic acid was observed with 5KZS protein. The volatile compound limonene was mostly active against PadR-like protein from *Listeria monocytogenes* (7WJP), a protein involved in various cellular mechanisms, including the regulation of efflux pumps and response to environmental stress. In this study, we focused on comparing the interactions of 4,5-di-O-caffeoyquinic acid with various target proteins, emphasizing its strong binding potential as presented in Table 3.

**Figure 1:**
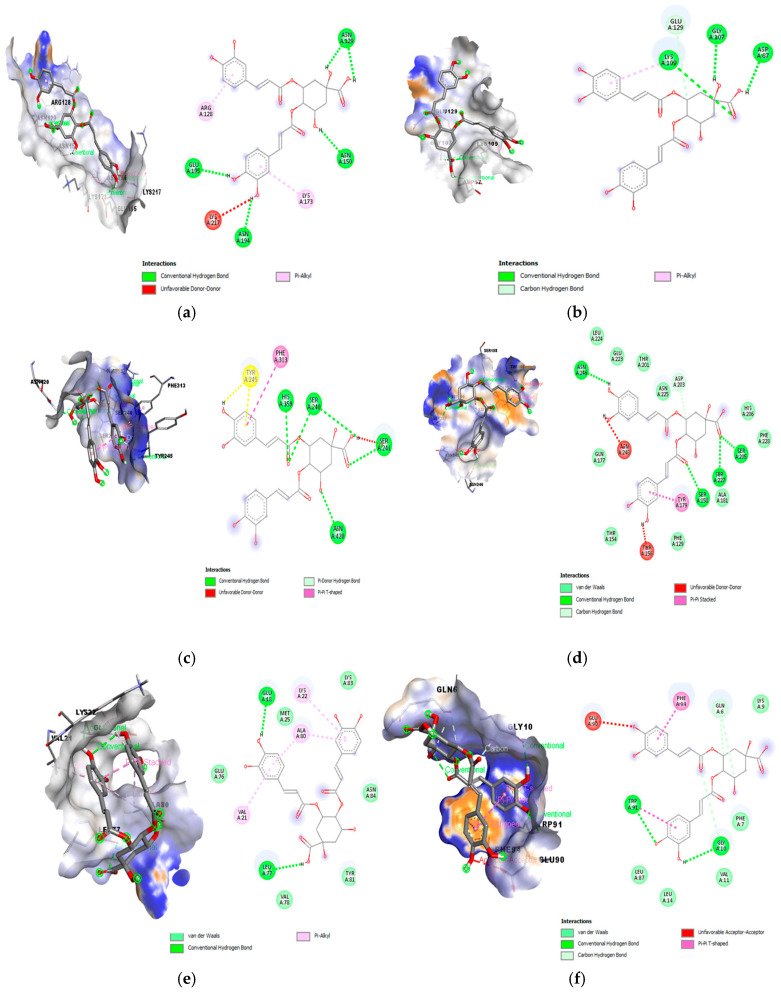
Two-dimensional and three-dimensional binding interaction of 4,5-di-O-caffeoyquinic acid toward the active site of (**a**) PDB: 1XEU, (**b**) PDB: 2WQV, (**c**) PDB: 4L3A, (**d**) PDB: 5KZS, (**e**) PDB: 6TIF, and (**f**) PDB: 7WJP.

The interactions types and binding positions of 4,5-di-O-caffeoyquinic acid with various receptors are illustrated in Figure 1. Key interactions with amino acid residues at the active site were visualized including hydrogen bonds, Van der Waals bonds, carbon hydrogen bonds and alkyl interactions, all of which play a crucial role in binding affinity and specificity, thereby contributing to *L. monocytogenes* inhibition. Notably, the 4,5-di-O-caffeoyquinic acid exhibited diverse interactions with 5KZS (Figure 1d) compared to other receptors, which explains its higher binding energy and enhanced ligand stabilization. Specific hydrogen bonds were identified with ASN246, SER158, SER227, SER205, and carbon hydrogen bond (ASP203), with 5KZS playing a key role in the binding process. Additionally, PHE129, ALA181, PHE228, HIS206, ASN225, THR201, GLU223, and LEU 224 displayed van der Waals contacts with 4,5-di-O-caffeoyquinic acid. These interaction types are reported to possess a key role in incorporating ligands in the active pocket but they showed lower interactions in comparison to the hydrogen bonds [34]. Unfavorable donor–donor interactions (ASN248 and THR156) and π-π stacked interactions (TYR179) were also observed, contributing to repulsion and influencing the spatial orientation of the ligand [37].

### 3.3. In Vitro Anti-Listeria Effect of LPE

**Figure 2:**
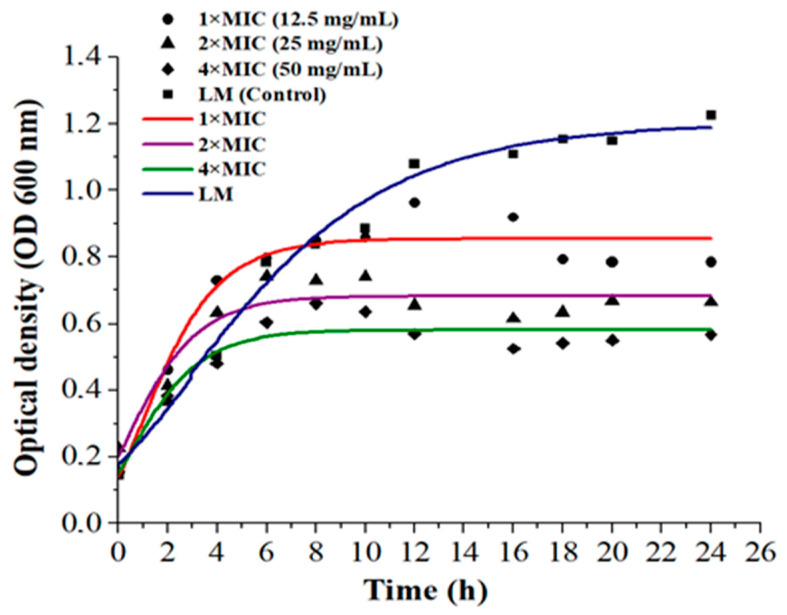
Effect of LPE at different concentrations (1, 2, and 4×MIC) on *Listeria monocytogenes* in vitro. LM: *Listeria monocytogenes,* MIC: minimum inhibitory concentration.

The antibacterial effect of LPE on *L. monocytogenes* was observed at a minimum inhibitory concentration (MIC) of 12.5 ± 0.4 mg/mL, demonstrating strong efficacy against this Gram-positive bacterium. The enhanced diffusion of phenolic acids through the cell membrane of Gram-positive bacteria and hyper-acidification of the plasma membrane are linked to phenolic acid dissociation [38]. In contrast, the lipopolysaccharide membranes of Gram-negative bacteria hinder such diffusion [39]. Figure 2 illustrates the growth curves of *L. monocytogenes* as influenced by LPE in broth media.

After 2 h of incubation, the antibacterial potential was found to be dose dependent. Compared to treated samples, the growth potential of the control medium (without LPE) was significantly higher, with a noticeable difference observed after 8 h of incubation (*p* < 0.0001) (Table S3). At LPE concentrations of 2×MIC and 4×MIC, no increase in bacterial density was detected after approximately 10 h of incubation. A reduction of 0.5 OD_600_ was recorded when 2×MIC concentrations of LPE were applied.

The application of 4×MIC concentration proved to be highly effective (*p* < 0.0001), resulting in a 0.7 OD_600_ reduction in *L. monocytogenes* density compared to the control after 24 h. In contrast, the optical density (OD_600_) of *L. monocytogenes* treated with 1×MIC concentration fluctuated between 8 to 20 h, showing a comparatively lower reduction. This effect aligns with findings by Konate et al. [40], who attributed similar outcomes to polyphenol-rich fractions. The antimicrobial activity of LPE is primarily referred to limonene, which diffuses through and increases the permeability of bacterial membranes [41], and phenolic compounds, as supported by the docking analysis. Bajko et al. [42] also reported a strong antimicrobial effect of O-caffeoylquinic acid against both Gram-positive and Gram-negative bacteria.

### 3.4. Anti-Listeria Effect in Fermented Milk During Storage at 4 °C

#### 3.4.1. Effect of LPE on *L. monocytogenes* Survival in Fermented Milk and Models Fitting

**Figure 3:**
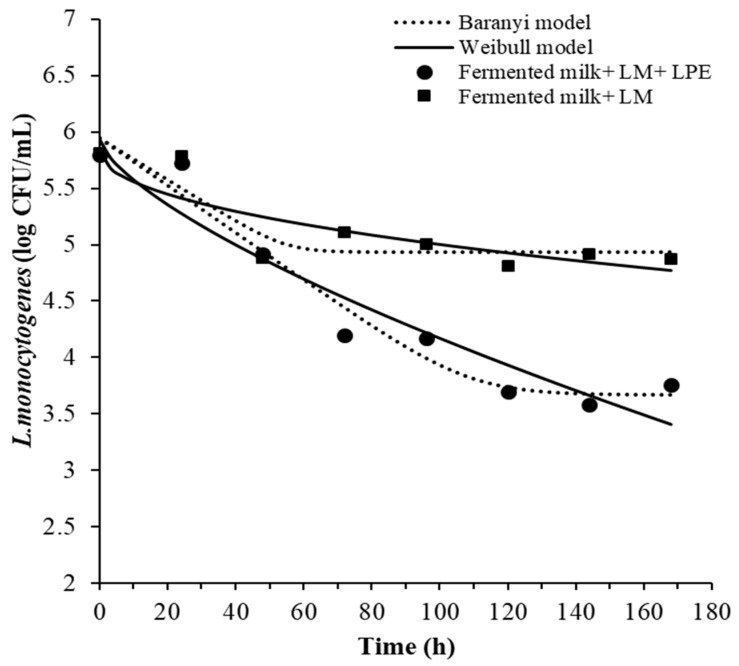
Survival of *L. monocytogenes* in fermented milk samples with 2×MIC of LPE (25 mg/mL) or without LPE during storage at 4 °C. LPE: lemon peel extract; LM: *L. monocytogenes*.

The survival curves of *L. monocytogenes* in fermented milk treated with LPE at 2×MIC concentration are shown in Figure 3. The concentration of *L. monocytogenes* decreased over the storage period in fermented milk samples with and without LPE. A reduction of 0.9-log of *L. monocytogenes* was observed in fermented milk samples without LPE, while a 2.04 log reduction was achieved in fermented milk samples containing LPE by the end of storage period. After 7 days of storage, the *L. monocytogenes* count in fermented milk with LPE (3.76 ± 0.07 log CFU/mL) was significantly lower (*p* < 0.001) than in fermented milk without LPE (4.88 ± 0.05 log CFU/mL).

The survival ability of *L. monocytogenes* in dairy products has been extensively studied [43]. It has been reported that *L. monocytogenes* can endure stress conditions, such as low temperatures and a wide pH range [44]. The kinetic parameters describing the behavior of *L. monocytogenes* were estimated using the Weibull and Baranyi and Roberts (without lag) models and are shown in Table 4.

These two models have been widely applied to describe microbial behavior and determine inactivation kinetics [44]. The applied models demonstrated acceptable goodness-of-fit parameters, with high regression coefficients (R^2^ ranging from 0.70 to 0.95) and low root mean square error (RMSE) values (0.19–0.279). According to the results, the shape parameter (β) of the Weibull model (0.5 and 0.7 < 1) (Table 4) indicated a slightly concave upward curve for both fermented milk samples. The model scale parameter (δ) in fermented milk containing LPE (44.09 h) was lower than that estimated for fermented milk without LPE (142.8 h). This parameter suggests a greater sensitivity of *L. monocytogenes* to LPE.

Based on the goodness-of-fit indices (R^2^ and RMSE), the Baranyi and Roberts model (without lag phase) was found to be the most accurate in predicting *L. monocytogenes* behavior. The R^2^ values for fermented milk without and with LPE were 0.7 and 0.9, respectively, using the Weibull model, and 0.78 and 0.95, respectively, with the Baranyi and Roberts model. The estimated parameters from the Baranyi and Roberts model showed that the maximum growth rate and the final cells counts were lower (Table S3) in fermented milk with LPE (−0.021 h^−1^ and 3.67 log CFU/mL, respectively). Numerous factors influence the behavior of *L. monocytogenes* in food products, including pH, water activity (aw), the presence of NaCl, and indigenous microflora. Additionally, storage temperature did not appear to impact the growth of *L. monocytogenes* due to its resistance to low temperatures. Previous studies have also demonstrated that lactic acid bacteria can inhibit *L. monocytogenes* growth by competition for nutrients and antagonism through the production of antimicrobial metabolites, like bacteriocins and organic acids [45].

#### 3.4.2. Lactic Acid Bacteria and Total Bacteria Counts

**Figure 4:**
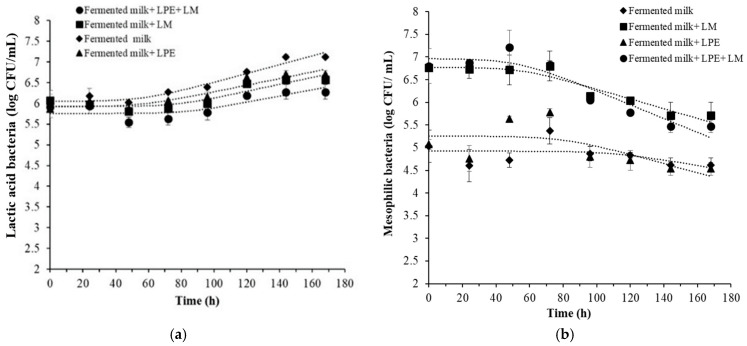
Curves of lactic acid bacteria (**a**) and mesophilic bacteria (**b**) in fermented milk, inoculated or not inoculated with *L. monocytogenes* and with or without LPE (2×MIC) during storage at 4 °C. LPE: *Citrus limon* peel extract; LM: *L. monocytogenes*.

The enumeration results obtained for lactic acid and mesophilic bacteria in the fermented milk samples are illustrated in Figure 4.

The growth of lactic acid bacteria increased during storage in all of the fermented milk samples (Figure 4a). The number of lactic bacteria was similar over 48 h of storage but increased after 72 h. The maximum bacteria loads were reached in fermented milk (7.12 ± 0.06 log CFU/mL) and in fermented milk with LPE (6.70 ± 0.04 log CFU/mL) compared to fermented milk samples with *L. monocytogenes*. The gradual increase in lactic bacteria counts was correlated with a decrease in *L. monocytogenes*, as these lactic acid bacteria were previously known to have anti-*Listeria* activity and can compete with pathogenic bacteria for nutrients and/or space [46].

Martín et al. [47] demonstrated the inhibitory effect of *Lc. Casei* against *L. monocytogenes* in soft-ripened “Torta del Casar” cheese. A reduction up to 5 log CFU/g, from an initial count of 7 log CFU/g, was observed during cheese maturation in samples supplemented with *Lc. Casei*. Regarding the growth behavior of the total microbial counts (Figure 4b), the results showed a stable level during the first 72 h, followed by a decrease, suggesting potential interaction or competition between pathogenic bacteria and other microorganisms. The behavior of mesophilic bacteria plays a crucial role in determining the acidity and shelf life of dairy products. It is therefore essential to monitor and control the dynamic interaction with microbial population to ensure the production of high-quality dairy products [48].

#### 3.4.3. Effect of LPE on pH and Acidity

**Figure 5:**
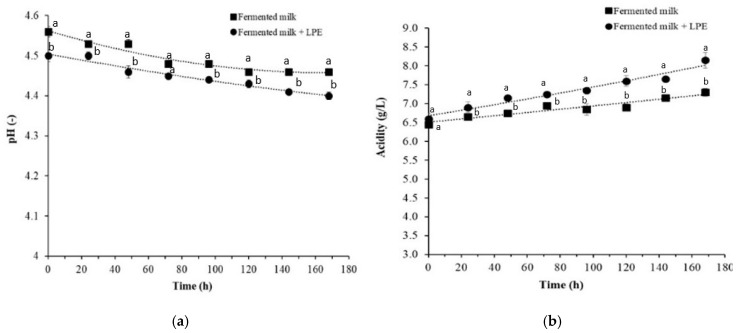
Changes in pH (**a**) and acidity (**b**) of fermented milk samples with or without lemon peel extract (2×MIC) during storage at 4 °C. Different letters indicate significant differences between samples (*p* < 0.05, Tukey’s test). LPE: lemon peel extract.

The results of pH and acidity are presented in Figure 5. A decrease in pH was observed during storage, with fermented milk containing LPE showing lower pH levels compared to samples without LPE (Figure 5a). Consistent with the pH results, the acidity was slightly higher in fermented milk with LPE (Figure 5b).

During storage, acidity increased from 6.45 ± 0.05 to 7.30 ± 0.10 g/L for control-fermented milk and from 6.60 ± 0.14 to 8.15 ± 0.21 g/L for fermented milk with LPE. The pH and acidity are crucial indicators of product quality and safety, particularly for their impact on pathogenic bacteria. The decrease in pH during storage can be attributed to post- acidification, which aligns with findings from previous studies. Wemmenhove et al. [49] reported that a lower pH in cheese was associated with higher concentrations of undissociated acid, which exhibits anti-*Listeria* effects.

#### 3.4.4. Organic Acids Profiles and Antioxidant Properties

The composition of organic acids of fermented milk samples are shown in Table 5.Eight organic acids were detected in the samples, with lactic acid being the most abundant. Organic acid levels were significantly higher in fermented milk (FM) compared to raw cow milk (RCM), particularly for lactic acid (RCM: 0.116 ± 0.003 g/L and FM at day 0: 11.30 ± 0.01 g/L). Propionic acid was present in FM but absent in RCM. Higher levels of organic acids were observed in FM with LPE compared to samples without the extract. During storage, the levels of lactic, formic, succinic, acetic, propionic, and malic acids decreased, likely due to metabolic reactions and the production of flavor-related compounds by lactic bacteria [50]. The supplementation of LPE in FM increased the amounts of organic acids, particularly lactic, succinic, acetic, and propionic acids. The higher organic acid content can also enhance the antioxidant properties of the product, as reported in previous studies [51].

The variations in total phenol and flavonoid contents, as well as the antioxidant activity of fermented milk during storage, are presented in Figure S1. Fermented milk with LPE exhibited higher total phenol and flavonoid contents compared to control samples (without LPE) (Figure S1a) and demonstrated greater antioxidant potential (Figure S1b). Control fermented milk samples exhibited lower antioxidant activity. In this study, organic acids, particularly lactic acid, along with antioxidant compounds from LPE, appeared to be the key factors contributing to the inhibition of *L. monocytogenes* growth in fermented milk.

## 4. Conclusions

In this study, the anti-*Listeria* effect of LPE was analyzed in silico, tested in vitro, and validated in fermented cow milk during cold storage. The Baranyi and Roberts model was the most accurate in predicting the decay kinetics of *L. monocytogenes,* confirming the inhibitory effect of LPE. The supplementation of fermented milk with LPE at a 2×MIC concentration reduced *L. monocytogenes* by 2.04 log CFU/mL and shortened the time required to achieve the first tenfold reduction of the pathogen. Additionally, the incorporation of LPE enhanced the antioxidant activity and total organic acid content of the fermented milk, primarily due to the presence of chlorogenic acid derivatives and limonene.

Further studies are needed to evaluate whether the addition of LPE affects the organoleptic properties of fermented milk or other dairy products to which it may be added. This evaluation is crucial for ensuring consumer acceptance of this anti-Listeria mitigation approach. Additionally, conducting a cost–benefit analysis of implementing this option is important, particularly in terms of potential price increases for LPE-supplemented milk. This analysis should also take into account the added value of reusing lemon peel in the process. Optimizing the extraction process to maximize bioactive compound recovery while maintaining affordability will be essential for its potential industrial application.

## Figures and Tables

**Table 1:** Volatiles compounds identified in LPE (percentage mean value ± standard deviation), retention times, and odor properties.

Compound	RT (min)	Area (%)	Odor Properties
α-Phellandrene	7.40	0.9 ± 0.1	Turpentine, mint, spice
Trans-β-Ocimene	7.568	4.7 ± 0.1	Citrus, herb, flower
Sabinene	8.550	0.370 ± 0.002	Pepper, turpentine, wood
β-Pinene	8.633	15.6 ± 0.2	Pine, resin, turpentine
6-Methyl-5-hepten-2-one	8.850	0.46 ± 0.03	Citrus
β-Myrcene	8.959	1.6 ± 0.1	Balsamic, must, spice
β-Cymene	9.792	0.6 ± 0.1	Solvent, gasoline, citrus
Limonene	9.924	68.7 ± 0.5	Lemon, orange
γ-Terpinene	10.634	6.8 ± 0.2	Lemon
2.5.5-Trimethyl-1-hexen-3-yne	13.956	0.42 ± 0.01	Spice
Geraniol	15.077	0.37 ± 0.01	Rose, geranium

Odor-related properties were identified from www.flavornet.org and www.thegoodscentscompany.com databases; RT: retention time.

**Table 2:** Individual phenolic constituents of LPE, retention times, [M-H]-m/z values, and concentrations (expressed in mg/g).

Compound	RT (min)	[M-H]-m/z	Concentration (mg/g)
Quinic acid	1.948	191	3.3 ± 0.1
Protocatehuic acid	7.087	153	0.031 ± 0.002
Epicatechin	19.137	289	0.0061 ± 0.0001
4,5-di-O-caffeoyquinic acid	22.770	515	10.9 ± 0.3
p-Coumaric acid	23.238	163	0.0133 ± 0.0003
Trans ferulic acid	25.978	193	0.0028 ± 0.0002
Rutin	27.814	609	0.205 ± 0.003
Hyperoside	28.860	463	0.014 ± 0.001
o-Coumaric acid	30.438	163	0.129 ± 0.004
Naringin	29.259	579	0.142 ± 0.003
Salviolinic acid	33.376	717	0.040 ± 0.001
Trans-cinnamic acid	35.648	147	0.003 ± 0.001
Naringenin	37.393	271	0.003 ± 0.001
Cirsiliol	39.055	329	0.0042 ± 0.0003
Cirsilineol	41.356	343	0.004 ± 0.002

**Table 3:** Binding affinity of selected phenolic and volatiles compounds against *L. monocytogenes* proteins.

Targets	LPE Compounds			
	Volatiles		Phenolic	
	β-Pinene	Limonene	Quinic Acid	4,5-di-O-caffeoyquinic Acid
1XEU	−4.3	−4.6	−5.2	−7.3
2WQV	−4.7	−5.1	−5.0	−7.1
4L3A	−4.7	−4.9	−5.2	−7.0
5KZS	−4.2	−4.7	−5.8	−7.4
6TIF	−5.4	−4.8	−4.8	−6.4
7WJP	−5.5	−5.6	−5.1	−6.3

**Table 4:** Kinetic parameters and fit quality of Baranyi Roberts and Weibull models applied to experimental data assessed in fermented milk samples.

Fermented Milk Samples	Models										
	Weibull					Baranyi Roberts (no Lag)					
	N_0_ (log CFU/mL)	β	δ (h)	RMSE	R^2^	N_0_ (log CFU/mL)	Maximum Growth Rate (h^−1^)	N_f_ (log CFU/mL)	Average of Total Reduction (log CFU/mL)	RMSE	R^2^
Fermented milk without LPE	5.85	0.5	142.8	0.23	0.70	5.94	−0.018	4.93	1.005	0.19	0.78
Fermented milk with LPE	5.93	0.7	44.09	0.28	0.90	5.95	−0.021	3.67	2.28	0.18	0.95

LPE: lemon peel extract; N_0_: initial number of *L. monocytogenes* in fermented milk (log CFU/mL); y_0_: initial number of *L. monocytogenes* in fermented milk (log CFU/mL); y_f_: final number of cells (log CFU/mL); µ_max_: maximum growth rate (h^−1^); average of total reduction (log CFU/mL); RMSE: root mean squared error; R^2^: regression coefficient; β: shape parameter of the Weibull model; δ: time required to have a first tenfold reduction of the *L. monocytogenes* counts (h).

**Table 5:** Organic acids concentration (expressed by g/L) in raw milk and fermented milk samples at day 0 and day 7 of storage.

	RCM	FM D0	FM D7	FM D0 + LPE	FM D7 + LPE
Lactic acid	0.116 ± 0.003 ^d^	11.127 ± 0.008 ^a^	11.30 ± 0.01 ^a^	10.34 ± 0.05 ^a^	11.64 ± 0.04 ^a^
Formic acid	0.692 ± 0.001 ^b^	1.738 ± 0.004 ^b^	1.66 ± 0.02 ^b^	1.959 ± 0.003 ^c^	1.886 ± 0.005 ^c^
Succinic acid	0.995 ± 0.004 ^a^	0.38 ± 0.01 ^c^	0.26 ± 0.05 ^de^	3.01 ± 0.15 ^b^	2.1 ± 0.1 ^b^
Acetic acid	0.13 ± 0.06 ^d^	0.292 ± 0.003 ^d^	0.284 ± 0.002 ^cd^	0.97 ± 0.04 ^d^	0.658 ± 0.005 ^d^
Citric acid	0.473 ± 0.002 ^c^	0.349 ± 0.005 ^cd^	0.41 ± 0.06 ^c^	0.446 ± 0.005 ^e^	0.316 ± 0.002 ^ef^
Propionic acid	ND	0.19 ± 0.03 ^e^	0.15 ± 0.02 ^e^	0.37 ± 0.05 ^e^	0.35 ± 0.01 ^e^
Malic acid	ND	0.02 ± 0.02 ^f^	ND	0.448 ± 0.002 ^e^	0.25 ± 0.02 ^f^
Oxalic acid	0.006 ± 0.001 ^d^	0.025 ± 0.005 ^f^	0.019 ± 0.001 ^f^	0.014 ± 0.001 ^f^	0.01 ± 0.00 ^g^
Total	2.41	14.12	14.08	17.56	17.21

RCM: raw cow milk; FM D0: fermented milk before storage (day 0); FM D7: fermented milk after 7 days of storage at 4 °C; LPE: lemon peel extract. Different letters indicate significant differences between samples (*p* < 0.05, Tukey’s test).

## Data Availability

The original contributions presented in this study are included in the article/Supplementary Materials. Further inquiries can be directed to the corresponding author.

## Supplementary Materials

The following supporting information can be downloaded at https://www.mdpi.com/article/10.3390/foods14040661/s1, Table S1: Receptor coordinates for selected PDB IDs from *Listeria monocytogenes;* Table S2: Different fermented milk samples prepared to study the effect of LPE against *Listeria monocytogenes* and the corresponding analyses; Table S3. Groupwise summary statistics based on Tukey (HSD) test for optical density and counts of *L. monocytogenes* for in vitro and in fermented milk studies; Figure S1: Total phenolic and flavonoid contents (a), and antioxidant activity (b) of raw milk and fermented milk during storage at 4 °C.
