# Supplementary material for: Assessment of the Anti-Listeria Effect of Citrus limon Peel Extract In Silico, In Vitro, and in Fermented Cow Milk During Cold Storage
Source: Foods. 2025 Feb 15;14(4):661. doi: 10.3390/foods14040661 (PMC11854709; doi:10.3390/foods14040661)
Supplement: Supplementary file 1 [file foods-14-00661-s001.zip › foods-3352442-supplementary.pdf]

Table S1. Receptor coordinates for selected PDB IDs from *Listeria monocytogenes*

Receptor Name	PDB ID	Coordinates
Internalin C from *Listeria monocytogenes*	1XEU	X :3.7223 Y :29.7415 Z :9.7343
Internalin domain of *Listeria monocytogenes* InlB	2WQV	X : -13.3138 Y :35.7183 Z :29.8891
Internalin K (InlK) from *Listeria monocytogenes*	4L3A	X :-37.5424 Y :-6.2882 Z :1.0213
Listeria monocytogenes internalin-like protein lmo2027	5KZS	X:-41.0715 Y:0.6215 Z:5.1216
ReoM- *Listeria monocytogenes*	6TIF	X :2.7593 Y :7.2733 Z :-17.5047
PadR-like protein from *Listeria monocytogenes*	7WJP	X : -1.1462 Y : 13.0548 Z : 9.2010

Table S2. Samples of fermented milk prepared to study the effect of lemon peel extract against *listeria monocytogenes* and the corresponding analyses

**Sample type**	**Code**	**Analyses**
Fermented milk without lemon peel extract and non-inoculated by *Listeria monocytogenes*	Fermented milk	▪ Physicochemical<br>▪ Microbiological<br>▪ Organic acids<br>▪ Antioxidant properties
Fermented milk added with lemon peel extract and non-inoculated by *Listeria monocytogenes*	Fermented milk +LPE	▪ Physicochemical<br>▪ Microbiological<br>▪ Organic acids<br>▪ Antioxidant properties
Fermented milk inoculated by *Listeria monocytogenes* without lemon peel extract	Fermented milk+ LM	▪ *Listeria monocytogenes* Kinetics<br>▪ Microbiological
Fermented milk inoculated by *Listeria monocytogenes* added with lemon peel extract	Fermented milk + LPE + LM	▪ *Listeria monocytogenes* Kinetics<br>▪ Microbiological

Table S3. Groupwise summary statistics based on Tukey (HSD) test for optical density and counts of *L. monocytogenes* for in vitro and in fermented milk studies

Time (h)	samples	0	2	4	6	8	10	12	16	18	20	24
In vitro	LM	0,144 b	0,368 d	0,505 c	0,785 b	0,838 b	0,887 a	1,082 a	1,109 a	1,157 a	1,147 a	1,227 a
	1×MIC	0,153 b	0,461 a	0,731 a	0,797 a	0,850 a	0,859 b	0,962 b	0,920 b	0,794 b	0,785 b	0,785 b
	2×MIC	0,228 a	0,414 b	0,632 b	0,742 c	0,730 c	0,741 c	0,657 c	0,615 c	0,634 c	0,665 c	0,664 c
	4×MIC	0,158 b	0,383 c	0,482 d	0,603 d	0,661 d	0,635 d	0,569 d	0,526 d	0,540 d	0,542 d	0,567 d
	Pr > F	**< 0,0001**	**< 0,0001**	**< 0,0001**	**< 0,0001**	**< 0,0001**	**< 0,0001**	**< 0,0001**	**< 0,0001**	**< 0,0001**	**< 0,0001**	**< 0,0001**
Storage time (h)	Samples	**0**	**24**	**48**	**72**	**96**	**120**	**144**	**168**			
In fermented milk	Fermented milk	5,809 a	5,785 a	4,886 a	5,108 a	5,007 a	4,810 a	4,917 a	4,877 a			
	Fermented milk +extract	5,796 a	5,728 b	4,917 a	4,200 b	4,167 b	3,692 b	3,584 b	3,756 b			
	Pr > F(Modèle)	0,664	0,014	0,856	< 0,0001	0,000	< 0,0001	< 0,0001	< 0,0001			

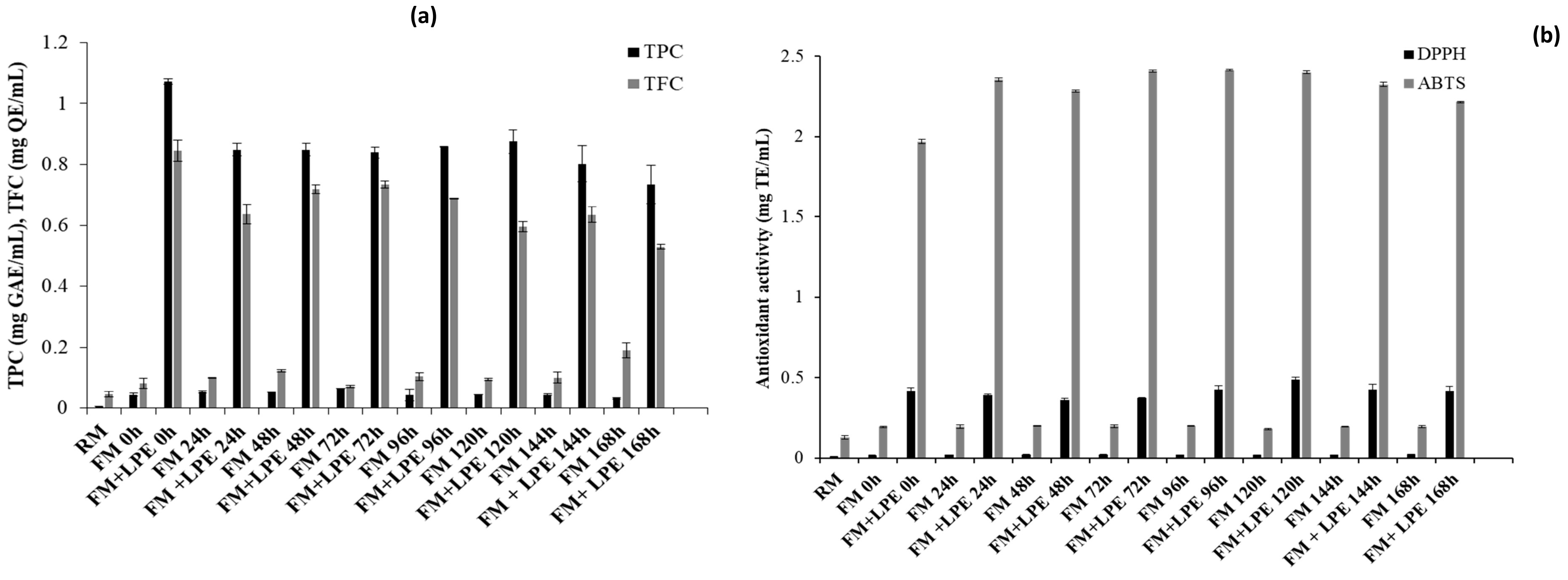

Figure S1. Total phenolic and flavonoid contents (a), and antioxidant activity (b) of raw milk and fermented milk during storage at 4 °C**;** *RM: Raw Milk; FM: Fermented milk; LPE: Lemon Peel Extract*
